# Trainee’s Perceptions on Simulation-Based Education and Its Impact on Transitioning to the Role of Medical Registrar: A Qualitative Study

**DOI:** 10.7759/cureus.72647

**Published:** 2024-10-29

**Authors:** Muhammad Molik

**Affiliations:** 1 Acute and General Medicine, York Hospital, York, GBR

**Keywords:** internal medicine training, postgraduate medical education, qualitative research, simulation in medical education, transition point

## Abstract

Background and aim

In 2019, a three-year internal medicine training (IMT) program replaced the two-year core medical training program in the United Kingdom, incorporating the first year of medical registrar (MR) training into the same curriculum. In light of the considerable evidence supporting the effectiveness of simulation-based education (SBE) and the challenges inherent in transitioning, the design and delivery of SBE are now ideally positioned to facilitate this transition process and assess its impact. However, trainees' perceptions regarding the effectiveness of SBE in the IMT curriculum for transitioning to the MR role are not well-documented. This study aimed to explore and examine trainees' views on SBE in the IMT curriculum to prepare them to transition to the role of the MR. It sought to identify the critical qualities and skills that IMT trainees believe SBE should address to enable them to successfully fulfill the MR role, as well as to assess the extent to which internal medicine year three trainees perceive that SBE in its current form within the IMT curriculum has prepared them for the role of MR.

Methods

This qualitative study employed focus groups to explore individuals' perspectives on SBE in the IMT curriculum and its role in preparing them for the transition to the MR position. Four focus groups were conducted, two with core trainees (CTs) and two with MRs. Sessions were recorded, transcribed verbatim, and analyzed using a thematic analysis approach.

Results

Eleven trainees participated in the study. Seven of them were CTs, with the remaining four in their first year as a MR. Six main themes emerged from the data, grouped into two main headings. The heading titled "Perceptions of SBE" included the themes "Valuable Teaching Tool," "A Squandered Resource," and "Avoid Over-Reliance." In contrast, the heading "Areas/Suggestions for Improving SBE" included the themes "Quantity and Scheduling," "Feedback," and "Draw on Others’ Experiences."

Conclusion

The value of SBE was recognized, alongside the vital role of high-quality feedback. Other medical specialties and industries were highlighted for their effective implementation of SBE, offering a possible standard for educators involved in the IMT curriculum to pursue. There was a perception that SBE did not adequately support the ongoing maintenance of skills, placing considerable reliance on real-world clinical practice to fulfill this need. The limitations inherent in SBE were acknowledged, and educators are encouraged to explore ways to optimize the use of all educational tools at their disposal, which may include the formal introduction of a shadowing period for trainees.

## Introduction

Internal medicine training (IMT)

IMT is a comprehensive medical training program designed for junior doctors in the United Kingdom (UK), aimed at equipping them to undertake the role of a medical registrar (MR) [1,2]. This comprehensive three-year full-time program comprises internal medicine year one (IMY1), internal medicine year two (IMY2), and internal medicine year three (IMY3). Replacing the two-year full-time core medical training (CMT) program, IMT follows the successful completion of foundation training and serves as a pathway to physician higher specialty training (PHST). The majority of trainees demonstrate foundation competence by successfully completing the two-year full-time United Kingdom Foundation Programme (UKFP).

The training deanery for each region organizes mandatory simulation (SIM) sessions [1]. In the Yorkshire and the Humber region, compulsory SIM sessions include Acute Simulated Core Medical Emergencies (ASCME), Communicating Medicine within Palliative Situations (CoMPaSs), and Internal Medicine Preparation for Registrars: Emergency Skills and Simulation (IMPRESS). Palliative medicine training is also provided through a series of sessions known as Extension of Community Healthcare Outcomes (ECHO).

Change from CMT to IMT

Before 2019, CMT was the postfoundation training program for medical specialties, preparing individuals for PHST applications. However, various issues emerged from this structure, prompting the decision to make changes.

As medicine becomes more specialized, there are concerns that newly qualified consultants may struggle to offer comprehensive care to an aging population with complex health requirements [1]. IMT aims to increase the number of consultants holding a general internal medicine certificate of completion of training, enabling more of them to participate in acute medical care. To further prepare future consultants, IMT now provides mandated experience in critical care, outpatient clinics, and geriatrics [1,2]. Core trainees (CTs) commonly experienced frustration with the "tick-box" nature of CMT. By replacing this with the capabilities in practice model, IMT helps to alleviate this burden [1,2].

CMT trainees were expected to attain membership in the Royal Colleges of Physicians of the United Kingdom (MRCP{UK}) before the end of their training program for a satisfactory Annual Review of Competency Progression (ARCP) outcome. IMT offers some leniency for those who have not obtained MRCP(UK) by IMY2, allowing them to continue their progression without meeting this requirement, with potential changes to their training and completion timeline determined by their deanery [1,2]. In contrast, CMTs risked missing out on PHST posts if they failed to meet MRCP(UK) passing deadlines, delaying career progression [1,2].

Although IMT increases the program length from two years to three years full-time (for those applying to group one specialties only), there has been a reduction in many PHST programs from five years to four years to compensate; however, for some specialties, e.g., cardiology, this has not been the case [1].

The IMY3 training year and becoming a MR

With the change from CMT to IMT, the transition to the MR role is now integrated into the same training program, aiming to provide additional support and supervision to trainees during this crucial period. The MR is next in line to the consultant, typically with a minimum of three years of postgraduate experience [3]. Recognized as a senior team member, the MR carries a wide range of responsibilities, including leading the acute medical on-call service and the acute medical assessment unit, acting as the primary referring physician for adult medical issues in the hospital and the community/general practice, addressing challenging situations with patients and families that other team members have not resolved, and leading acute medical emergencies and cardiac arrests [3,4]. This significant step up, particularly in the areas of management and leadership compared to their previous role as a CT, represents a major transition point in medical training [3].

Transitions

While there is a wealth of literature exploring the progression from non-clinical to clinical or from registrar to consultant, there is a significant lack of research when it comes to the transition from CT to registrar [3,5]. Nonetheless, the little literature that does exist indicates that this transition is challenging, involving periods of uncertainty, heightened expectations in management and leadership, and reduced support [3,6].

Simulation-based education (SBE)

In medicine, various forms of SBE are utilized to train individuals in emergency presentations, non-technical skills, and procedures. Cook et al. in 2011 performed a systematic review and meta-analysis which demonstrated that SBE at the postgraduate level consistently led to enhanced educational outcomes in various clinical topics and types of SBE, compared to no educational intervention as control [7].

In light of the extensive body of literature surrounding SBE, a report was published by the joint JRCPTB/HEE Expert Group on Simulation in Core Medical Training [8]. This study involved a thorough review of existing literature and gathering expert insights on optimal practices for incorporating SBE into the CMT curriculum which resulted in the recommendation below. This recommendation has been integrated into the IMT curriculum.

*“That all essential and desirable practical procedures listed in the CMT curriculum should be taught by simulation as early as possible in year one, with further simulation teaching involving human factors and scenarios training carried out in either year one or year two. The latter should also include refresher training for procedural skills where necessary"* [8].

Knowledge gap

Given that the transition to the MR role now occurs within the same curriculum, and considering the extensive evidence in the literature on the effectiveness of SBE and the challenges of this transition, the structure and content of SBE delivery are optimally positioned to support the transition process and evaluate its impact. However, the perceptions of trainees regarding the effectiveness of current SBE delivery in the IMT curriculum for transitioning to the MR role are not well-documented, emphasizing the need for additional knowledge in this specific area.

## Materials and methods

Theoretical stance

This study aimed to explore the lived experiences of IMT trainees, their perceptions and perspectives of SBE, and their potential influence in their transition to the role of MR. This is a relativist and subjective stance that acknowledges the existence of multiple realities and the diverse ways in which individuals experience them, where no one "ultimate truth" or "correct" way of knowing exists [9]. Therefore, the choice was made to adopt interpretative, or hermeneutic phenomenology, as the primary approach with focus groups (FGs) chosen as the primary method of data collection.

Participants, sampling, and recruitment

IMT trainees residing in the Yorkshire and the Humber region were extended an invitation to participate in this study. Invitations were distributed via email. Additionally, invitations were extended at various regional IMT teaching sessions through Zoom (San Jose, CA: Zoom Video Communications), held between February 2024 and April 2024.

Convenience and criterion sampling were employed for participant recruitment, where IMT trainees who expressed interest were selected for inclusion in an FG on a first-come-first-serve basis. Eleven participants in total were recruited. In the FG transcripts, the seven CTs were anonymized and identified as CT1, CT2, CT3, CT4, CT5, CT6, and CT7, while the four MRs were anonymized and identified as MR1, MR2, MR3, and MR4. Data obtained from the questionnaires showed a fairly balanced distribution between both genders. Additionally, there was representation from both domestic and international graduates, with all participants having undergone some form of SBE during their postgraduate training. A comprehensive summary can be found in Table 1. Unfortunately, one FG participant (an IMY1/IMY2 trainee) did not complete the questionnaire, while one individual (an IMY3 trainee) who completed the questionnaire did not participate in a FG.

**Table 1:** Demographic data for trainees who completed the questionnaire. IMY1: internal medicine year 1; IMY2: internal medicine year 2; IMY3: internal medicine year 3; PMQ: primary medical qualification; UK: United Kingdom *1 IMY1/IMY2 trainee who participated in the focus group did not complete the questionnaire.

Variables	Number of trainees (%)
	Total = 11*
Gender	
Male	6 (55)
Female	5 (45)
Current training grade	
IMY1	3 (27)
IMY2	3 (27)
IMY3	5 (45)
Place of PMQ	
UK	4 (36)
Outside the UK	7 (64)
Simulation training received (undergraduate level)	
Yes	8 (73)
No	3 (27)
Type of undergraduate simulation training received	
Procedures	8 (73)
Emergency presentations	8 (73)
Non-technical skills (human factor skills)	5 (45)
Simulation training received (postgraduate level)	
Yes	11 (100)
No	0 (0)
Type of postgraduate simulation training received	
Procedures	11 (100)
Emergency presentations	10 (91)
Non-technical skills (human factor skills)	7 (64)

Data collection

Four FG sessions, two comprising IMY1/IMY2 trainees (CTs) and two comprising IMY3 trainees (MRs) were undertaken. Regarding the composition of FGs, MRs were separated from CTs to prevent perceived hierarchies and significant power imbalances within groups inhibiting free discussion [10].

The researcher took on the role of facilitator for all four sessions. For each of these sessions, a set of facilitator questions (appendix A) were prepared. To ensure the quality of these questions, they were reviewed by a medical education research colleague who possesses experience in conducting qualitative research in the field of medical education. Sessions were limited to a maximum timeframe of 60 minutes, which is considered the minimum optimal duration while being mindful of not imposing any unnecessary burden on the participants' time [10]. Demographic data and participants' previous experiences with SBE were collected through a concise questionnaire (appendix B), which participants could complete before or after the FG sessions.

Data analysis and researcher

Data were analyzed using thematic analysis (TA) with an inductive approach. The widely adopted version of TA as outlined by Braun and Clarke utilized the following six-step process (familiarization of data, initial coding, generating themes, reviewing themes, defining/naming themes, and interpretation/reporting) [11].

TA is aimed at comprehending the experiences, thoughts, and behaviors of participants through the analysis of qualitative data. This method entails closely examining the dataset to identify, analyze, and document recurring patterns [11]. It encompasses the interpretation of data through the selection of codes and the construction of themes, in addition to its role in data description [12].

Regarding the identification of themes, an iterative, inductive approach was taken. This entails coding the data without attempting to impose it into an existing framework or any preconceived notions from the researcher [13-15]. Therefore, this type of TA is driven by the data itself [11]. The choice of TA in this study was due to its relative simplicity in learning and application, wide usage, and suitability for researchers with little or no experience [11]. It does not require the in-depth understanding other qualitative methods demand. The researcher who conducted the FGs and analyzed the data is a postgraduate Medical Education student currently undertaking the Masters in Medical Education (MMedEd) course at Newcastle University.

Ethical approval

Full ethical approval was received from the Newcastle University Faculty of Medical Sciences Ethics Committee (#2681/41350). Before signing the consent form, individuals showing interest were given a participant information sheet. This outlined the purpose of the study, what it involves, what happens to the data collected, ethical approvals obtained, and sponsors. Contacts were listed if further information was required and clearly stated that participants could withdraw without consequence at any time. It was provided in advance to signing the consent form, with appropriate time given for participants to read and ask questions.

FG sessions were recorded via Zoom. Recordings on Zoom automatically produced transcripts which were stored securely as approved by Newcastle University. Transcripts were manually proofread and checked for errors. Personal details were removed and participants were anonymized. Recordings were then subsequently deleted.

## Results

**Figure 1:**
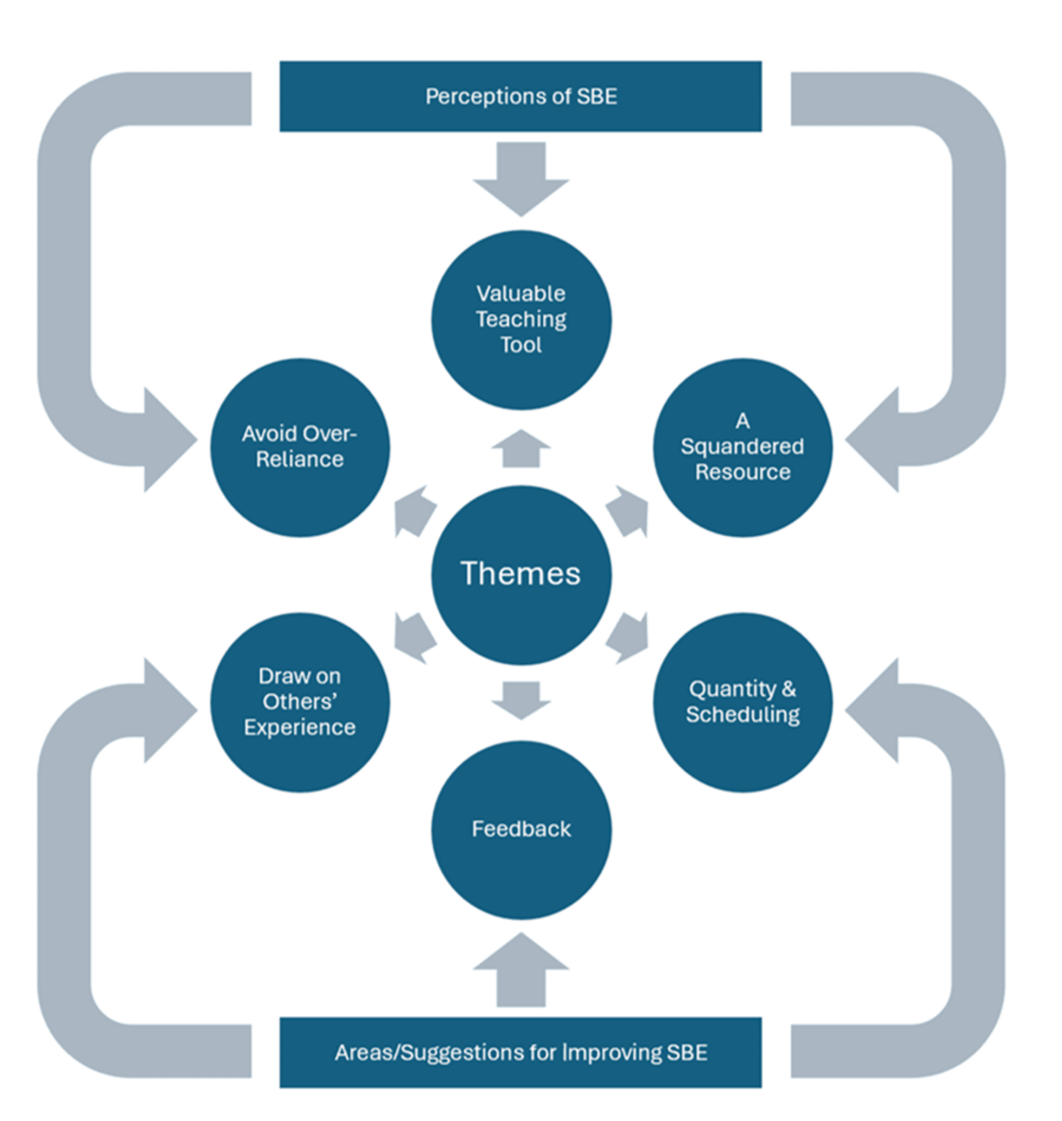
Themes relating to the perceptions of SBE (top) and themes relating to areas/suggestions for improving SBE (bottom). SBE: simulation-based education

Six key themes were identified and grouped under the headings "Perceptions of SBE" and "Areas/suggestions for improving SBE" (Figure 1). Factors identified for each heading and theme are outlined in Tables 2, 3.

**Table 2:** Factors identified for the heading "Perceptions of SBE." ARCP: Annual Review of Competency Progression; CBD: case-based discussion; CoMPaSs: Communicating Medicine within Palliative Situations; ICU: intensive care unit; IMPRESS: Internal Medicine Preparation for Registrars: Emergency Skills and Simulation; IMT: internal medicine training; MDT: multi-disciplinary team; MR: medical registrar; RWCP: real-world clinical practice; SBE: simulation-based education; SHO: senior house officer; SIM: simulation; US: ultrasound

Theme	Factors	Participant quotes
One - Valuable Teaching Tool	SBE is beneficial	“…simulation is the most effective and efficient learning in sort of teamwork, acute, clinical and emergency stuff, as well as those like challenging conversations…” [MR2]
		“…the CoMPaSs course, …escalation decisions and end of life conversations and dealing with sort of difficult patients, that was a very useful day.” [CT1]
	Session structure	“…gave us the opportunity to see different cases, talk through them at the end, and then, like, discuss it amongst ourselves, and then with the trainer as well.” [MR4]
		“…it helped reinforce whatever good habits I’ve tried to develop. It helped to actually, formally tell me it's like, yes, what you're doing is actually correct, …” [MR2]
	Safe and secure environment	“…it speeds up the rate at which you learn…because you’re put in a safe situation where you could basically do whatever, you could do whatever you want, you can absolutely get it totally wrong, but it's okay in that situation.” [MR2]
	Human factor skills (non-technical skills)	“It's useful to do simulation training to get exposure to all the human factors and stuff. We did that recently on IMPRESS.” [MR1]
		“…I found them really useful, particularly the non-technical skills one.” [MR3]
	Procedural skills	“…it feels right for the procedures, like I think you know, one course and then get practicing.” [CT2]
		“…helps you with the techniques.” [CT4]
	Cognitive framework	“…SIM obviously gives you a structure in your head.” [CT1]
		“…all these acute scenarios that I think, SIM, you know, it can help you have a bit of a kind of flow chart in your head for how to manage something.” [CT1]
	Training program/ePortfolio requirements	“…the fact that we got CBDs after it was it kind of made it more appealing because we all need things signing off on our portfolios.” [MR4]
Two - A Squandered Resource	Limited availability/access to resources/sessions	“…they don't have enough number of, er, manikins as well as I think the lecturers and teachers.” [CT1]
		“…pulling the ICU registrar, pulling Cardiac Outreach, pulling the MR to do a simulation, er, can be difficult technically.” [MR3]
	High IMT trainee/SBE session ratio	“…there are a lot of people, so not everyone gets a chance to do the SIM by themselves, because they pair up, or it's alternate, or either you do more than the other one.” [CT2]
	Limited leadership opportunities	“…not everyone has the opportunity to lead the scenario and do it.” [CT2]
		“…if you're going to do a SIM teaching session, each person should have to do a scenario. It's all very well watching someone, isn't it? But I think, unless you're in the situation and engaging your brain like you don't learn as much.” [CT1]
	Difficulty accessing non-mandated SBE courses	“…a post complication procedure course in the hull, which is very difficult for everyone to get in as IMT.” [CT2]
	Sessions neglect advanced knowledge/skills/behaviors	“…day to day MR job - some skills are necessary but not part of our SIM/ARCP as for example - US-guided cannulation, joint aspirations, etc.” [CT7]
	Recruit facilitators with prior experience as MRs	“…they could create SIMs that they've been involved with, as you know, junior MR’s for us.” [CT1]
	Recruit more MDT members from RWCP	“And utilize more members of the multidisciplinary team?” [Facilitator] “Yeah, the, the team you'd actually likely be working with and see how that works out in real life.” [MR3]
		“I think it will be enriching, with the team that you work with, …” [MR3]
	More local SBE sessions/facility access	“…I think going forward, it would be helpful to localize simulation. Sometimes we do these courses, erm, elsewhere with a different team…it’d be helpful to localize the simulation days.” [MR3]
Three - Avoid Over-Reliance	SBE has its limitations	“…there's some limitation because we know that it's not actual human.” [CT2]
	SBE cannot fully represent RWCP	“…if a patient is unstable, and you need to do like a chest drain, it’s way different than if it's a one-off simulation course on a mannequin. So, it's not real life.” [CT4]
	SBE sessions can be intimidating/overwhelming	“It can be intimidating if you have not done it before, …” [MR2]
	RWCP has a vital role	“…when it comes to emergency situations, I think everybody has to have some, erm, you know experience, see that this can go wrong, and what you do in that situation.” [CT5]
	RWCP has drawbacks	“…when we do go to any, er, any arrest calls, etc, as an SHO, we still end up waiting to be told what to do…we aren't put in the leadership role unless nobody else is around.” [MR4]
	Formalize shadowing	“…I don't know whether simulation is a solution to it. I wonder whether coaching is, perhaps, maybe one-to-one shadowing. Planned shadowing, perhaps, and maybe some simulation, holding the bleep, as people have suggested in the past. But all that is, is quite informal. Maybe formalize in this process, …” [MR3]
	Utilize all available teaching tools	“I feel there's benefit to repeating simulation regularly, but it then needs to have some sort of follow up with a continuous learning process in practice, in, you know, like in the real world, in a way. So use the simulation to get you up to speed. Get the learning, and the opportunity to do things wrong and get the right feedback, and then have a continuous learning process with things like coaching.” [MR2]
		“…with simulation, you can learn a lot faster and in a better situation. But it then needs to be followed up with a continuous learning process in the real world, and we said direct observation, so whether it be a very senior registrar being on the same take as the new registrar. But the new registrar has the role and responsibility than the more senior registrar's job is to observe and feedback.” [MR2]

**Table 3:** Factors identified for the heading "Areas/suggestions for improving SBE." CoMPaSs: Communicating Medicine within Palliative Situations; ECHO: Extension of Community Healthcare Outcomes; Gynae: gynecology; ICU: intensive care unit; IMT two: internal medicine training (year) two; IMT three: internal medicine (year) three; Med Reg: medical registrar; NHS: National Health Service; Obs: obstetrics; SBE: simulation-based education; SIM: simulation; UK: United Kingdom

Theme	Factors	Participant quotes
Four - Quantity and Scheduling	Increase number of SBE sessions	“…amount of simulation you feel you need more…” [Facilitator] “Exactly. Yes, I feel like we need more.” [CT6]
		“…more simulation would help us with our confidence and skills.” [CT6]
		“Yeah, I agree we should get more simulations.” [MR2]
	Increase number of acute SBE sessions	“I don't think we've received enough acute SIM scenario training, personally.” [CT1]
		“…if you got more, more complex acute scenarios, then it would prepare you for IMT three.” [CT3]
	Increase number of communications SBE sessions	“Clinical communication skills are good as covered in CoMPaSs or ECHO. More will be helpful.” [CT7]
	Schedule more SBE sessions prior to the transition	“…we don't become Med Reg’s straight after, cause we've still got IMT two, and we don't have a lot of simulation during IMT two,…” [MR4]
	Schedule SBE sessions regularly	“…weekly, fortnightly or even monthly sort of basis, just locally, you know, in small groups that would be very useful, I think. Making it more of like a regular thing.” [CT1]
Five - Feedback	Importance of regular high-quality feedback	“If someone judge you and give you feedback consistently, that would be really good in becoming IMT three.” [CT2]
		“…the instructors are, I think, the one I had in Doncaster, *****, was really helpful, and she gave very good feedback.” [CT6]
	Feedback can be inadequate	“Feedback part was not focused in many of them.” [CT7]
		“…feedback can be better… [MR3]
	Poor feedback prevents identifying areas of improvement/concern	“…you might have someone particularly who's not doing very well, and then they find themselves in trouble eventually. And then, if you retrospectively look back, that actually, all of their supervisors have been unhappy, and every course leaders been unhappy. But no one was, felt confident enough to give them the constructive feedback to say, well, this wasn't good enough, and this is how we might improve it,…” [MR2]
		“…when you're handing over to, you know, to someone, to ICU, or anesthetics in real life, they don't have to pick on every mistake that you do…They just accept kind of whatever kind of handover you give really. Unless they're really unhappy with the handover...” [MR2]
	Factors driving lack of quality feedback	“…I have always put it down to it, being cultural. I think you, in my own culture, things are quite upfront, and you're told as it is, irrespective of how you feel.” [MR3]
		“I think the person who's feeding back, is concerned about giving negative feedback and upsetting them.” [MR2]
		“…it's just easier to give positive feedback and move on. [MR2]
		“…trainers need to be trained on how to give feedback properly because it's very difficult. I think in these communication courses I think the most difficult aspect of the whole thing is the person who is observing, being able to give constructive feedback, and at times when appropriate negative feedback. Because it's too easy to be like, yeah, that was good, great, well done.” [MR2]
	Simulated patients offered better feedback	“…a lot of the simulated patients they had were professional simulated patients or patients with lived experience. Who was not shy about telling you what they thought, and because they were non-medical…it was very much on tone, particular words, language, body language, those elements. It was like, they would give good quality feedback on them.” [MR2]
		“For the patients, you know, there, there's a reason they volunteered to do that course is probably because they either had an excellent experience or a really bad experience, and either way they want things to be good and improve.” [MR2]
Six - Draw on Others’ Experiences	Learn from other industries	“So you think that other industries tend to utilize the tool far more effectively to create far more efficient teams?” [Facilitator] “Without a doubt. They use simulation a lot more.” [MR3]
	Aviation industry and SBE	“…if you look at, we compare ourselves in terms of high-risk jobs, you compare to things like aviation, and simulation is heavy in aviation. It’s probably where we need to get to, to get, all really well-drilled, oiled teams that work together like clockwork.” [MR3]
	SBE and other medical specialties	“It's well recognized by the surgical training program that more simulation makes better trainees because it gives them an opportunity to practice. And then, in effect, the faster they can become independent. Once they're independent, then they can continue to learn, is, they're more confident…” [MR2]
		“…in Obs and Gynae,…they do like a hybrid SIM, where they do it in person, in the hospital, and they pick a day when the rota looks good, and then they basically just set up, as some sort of SIM in one of the rooms, and just press the emergency buzzer. So then it's like in hospital,…” [MR2]
		“…that's really good for Obs and Gynae because they have, like, several emergencies a day, and some of them are, but some of them are quite rare, so they practice them.” [MR2]
	Diverse views of the NHS workforce	“I think we talk about culture but, the NHS is the most multicultural part of the entirety of the UK. And I think taking elements from other people's cultures,… I think that's what we need to learn and improve.” [MR2]

Perceptions of SBE

Theme One: Valuable Teaching Tool

The majority of participants recognized the value of SBE and acknowledged it as a high-quality educational resource. Several trainees mentioned that SBE courses were among the most beneficial courses they had taken as a doctor, with some specifically highlighting SBE courses they had attended within their IMT program.

“ASCME is one of the best courses I attended. Erm, it’s really useful.” [CT2]

The structure of SBE sessions was one aspect that significantly contributed to enhancing its educational value. By simulating certain elements of real-world clinical practice (RWCP), including monitors, paperwork, phone calls, and interactions with clinical staff, participants are given the opportunity to assume a leadership position in a scenario typical of RWCP. Subsequently, they can reflect on their performance and engage in peer discussions. Trainees were eager to evaluate their clinical practice and enhance their leadership skills, both of which are important qualities to possess when assuming the MR role. Trainees were eager to evaluate their clinical practice and enhance their leadership skills, which are important when assuming the MR role.

In addition to their own participation, by observing their colleagues handle familiar situations, they were able to witness both effective and ineffective management of these scenarios. These observations, followed by discussions and consensus-building with peers and facilitators regarding key learning points, help participants feel better equipped to manage similar encounters in clinical practice than they would otherwise.

This session structure further benefits participants by establishing a safe and secure environment to facilitate the acquisition of knowledge, skills, and behaviors. Within this secure environment, individuals can experiment and learn from errors without jeopardizing the safety of others, allowing for the refinement and acknowledgment of the correct approaches to take in RWCP.

“…it's a really good opportunity to make some mistakes, and then, you know, based on that, you hopefully will not repeat them again in your practice.” [MR2]

Through this session structure, participants were afforded the opportunity to address, refine, and improve their human factor skills, also known as non-technical skills. The importance of mastering these skills was acknowledged by participants as a fundamental necessity for effectively undertaking the role of the MR. These skills encompass making decisions regarding appropriate escalation, conducting end-of-life discussions, and honing communication skills with patients and their families.

Participants emphasized the importance of grasping fundamental aspects of procedural skills before practicing in healthcare settings, they feel it is inappropriate to carry out procedures in RWCP without any prior experience, even under supervision. However, they recognized the need for repeated practice on actual patients for genuine proficiency.

Additionally, for approaching acute medical scenarios in RWCP, SBE has been commended for its ability to provide a cognitive framework for individuals. Through this, participants feel more capable of achieving optimal outcomes in RWCP by managing critical and time-sensitive situations more efficiently and comprehensively. This framework provides participants with a systematic and intentional approach to use when confronted with feelings of stress, anxiety, and/or uncertainty, where maintaining clarity of thought and taking appropriate action - key attributes and expected qualities of an MR - becomes much more challenging.

Finally, meeting the training program and ePortfolio (London, England: National Health Service) requirements for facilitators and trainees was warmly welcomed. With willing and engaged assessors readily available, scenarios typically required for ePortfolios, and time set aside for performance assessments, all the essential elements are conveniently in place for individuals to take advantage of, fulfilling the mandatory requirements expected of them by their training programs. This opportunity was also suggested as a possible incentive to encourage appropriate individuals to get involved with the running and designing of SBE sessions.

“…they could maybe help facilitate it, and then they'd get, you know, teaching assessments signed off…” [CT1]

Theme Two: A Squandered Resource

Participants believe that SBE, despite acknowledging its value, is not being utilized to its full potential to provide them with all the benefits that it is thought to offer. Various reasons were cited for the perceived inadequacies in current SBE delivery in the IMT curriculum.

An important issue is scarcity of resources. Participants were aware of the challenges that organizers face in funding the required material resources for successful SBE sessions, as well as in finding suitable personnel to participate in and facilitate these sessions. This scarcity is likely the most significant factor contributing to the issues raised by participants regarding the current SBE structure in the IMT curriculum. Numerous suggestions for improving SBE delivery are rooted in addressing this specific issue in various ways. One of these is the perceived high number of IMT trainees participating in any given SBE session. This high trainee/session ratio, seen as excessive by participants, is likely due to the region's efforts to meet mandatory SBE requirements with limited resources.

“…there are more candidates compared to the amount of facilities that they can provide.” [CT2]

Consequently, issues arise such as trainees not always being given the opportunity to lead scenarios. Effective leadership is an essential aspect of the MR role, and participants recognize that they need to possess this skill before making the transition. Another issue lies in accessing non-mandated SBE courses due to limited places and the first come first serve policy. Participants recognize the value of undertaking SBE sessions, as previously outlined, and show a willingness to engage in sessions beyond those mandated by their training programs. Nevertheless, many struggle to sign up, especially with busy schedules that may prevent them from staying informed about available courses in a timely fashion, resulting in missed opportunities. The fact that participants are actively seeking out extra SBE sessions suggests that they feel the number of mandated sessions is not enough. Additionally, many feel that SBE delivery in the IMT curriculum often fails to adequately address the advanced competencies required for an MR to effectively carry out their duties.

“…as my colleague mentioned is like beginners, beginner level teaching.” [CT4]

Participants feel some key skills, such as ultrasound-guided cannulation, and joint aspiration are absent, and some find SBE sessions too rudimentary to be beneficial. Many participants therefore want to ensure the recruitment of SBE facilitators who have personal prior experience as MRs. These individuals can then develop sessions based on their past encounters in RWCP and are therefore deemed more likely to create scenarios that offer the complexity that participants are seeking.

Participants suggested involving various multi-disciplinary team (MDT) members from RWCP in SBE sessions for enhanced effectiveness. Trainees often take these roles in scenarios, while a colleague takes the lead. Nevertheless, encouraging MDT members to participate and embrace their individual roles would allow SBE sessions to more closely resemble RWCP. It would enable healthcare teams to engage in collective practice, discussion, reflection, and rapport-building. This approach can enhance team dynamics and foster collaboration, ultimately resulting in more effective and efficient healthcare teams that positively impact patient care.

Conducting these SBE sessions locally would facilitate this opportunity, providing a more realistic and beneficial learning experience to not only doctors but also other allied health professionals who could benefit from what SBE has to offer. Finally, numerous participants are mindful of their role at this stage of their career as independent and self-directed learners. They feel utilizing local teaching facilities and simulation equipment post-SBE courses to further hone their procedural skills and re-enact scenarios with peers would prove advantageous.

“…I'd like to be more like, right, there's a SIM room for procedures, you've done your course. This is now available for you, say, for the rest of the year. Walk up at whatever time you're free, and go practice…” [MR1]

Theme Three: Avoid Over-Reliance

Despite the aforementioned advantages associated with SBE, many participants acknowledged its limitations and emphasized that it cannot solely prepare them adequately for transition.

“…I think SIM helps, but it, you know, it doesn't, it won't, it can't fully, can't fully prepare you to what's going to happen.” [CT1]

More specifically, it was recognized that SBE is unable to encompass all the intricate complexities found in RWCP. For instance, the distinction between practicing a procedure on a mannequin in a controlled educational setting and carrying it out in a real-life acute situation with an actual patient, where help from a colleague capable of performing the procedure may not be readily available, and other urgent tasks demanding attention. These two scenarios are not comparable.

Additionally, it was acknowledged that SBE sessions can be overwhelming and intimidating for participants. This is understandable, as these sessions often involve many of their peers observing and evaluating their performances. The expectation to meet not only their own standards but also those of others can result in a less-than-optimal learning experience.

Medical professionals are recognized for upholding high standards of themselves, resulting in elevated stress and anxiety levels when tasked to perform. This is despite SBE facilitators' attempts to emphasize that SBE sessions are intended for learning, not for assessing competence.

“I think I panic, and I don't learn if I think lots of people are watching me or judging me.” [CT1]

Participants recognized the vital role that RWCP plays, especially in light of the limitations of SBE. However, they have also pointed out certain drawbacks within RWCP itself. One such drawback is the lack of opportunities for trainees to develop their leadership skills. Trainees have noted that during acute emergency situations, like cardiac arrest scenarios, the senior colleague automatically assumes the leadership role, whether it is the MR or consultant.

IMY1 and IMY2 trainees find it challenging to take on leadership in emergencies as they are unfolding, and reluctant to ask their seniors beforehand. The current shift pattern hinders the development of strong working relationships, which could help trainees feel more at ease asking their senior colleagues to take on such responsibilities, and for senior colleagues to feel more reassured from prior experience working with these individuals to delegate such responsibilities to them. Another drawback is participants struggle to apply SBE skills due to limited practice opportunities in RWCP, hindering the development of confidence and mastery through repetitive practice.

“…the thing is, if you don't do it again, or if you don't practice again, then, erm, it's not necessarily very helpful.” [CT4]

Some participants expressed a strong interest in formalizing a structured mentoring program, also known as shadowing, into the curriculum. This would involve an IMY2 trainee, during the concluding months of their training year, assuming the responsibilities of an MR (carrying the necessary bleep and performing MR duties) while under the supervision of an on-site MR who can provide support when required. Although some trainees have previously taken the initiative to establish similar arrangements, this has typically been trainee-driven and dependent on the willingness and availability of their MR colleagues, which may not always be the case at all hospital sites.

Despite the notion that more SBE could somewhat alleviate the scarcity of opportunities in RWCP, the prevailing sentiment emphasized the importance of effectively utilizing all the various teaching tools available, such as SBE, RWCP, shadowing, and high-quality feedback, to provide trainees with the best platform to effectively transition. This belief stems from the understanding that a continuous learning process is essential for adequately preparing for transition, which cannot be accomplished solely through SBE.

Areas/suggestions for improving SBE

Theme Four: Quantity and Scheduling

Concerns have been raised by participants regarding the quantity of SBE sessions they are receiving. Many trainees believe that the current number of sessions is insufficient and are advocating for an increase in mandated sessions within the IMT curriculum.

Specifically, numerous participants believe that there is a shortage of sessions focused on handling acutely unwell patients or tackling emergency situations. CTs in particular emphasized increasing these types of SBE sessions in IMY1 and IMY2 before making the transition. This highlights the apprehensions participants have regarding their preparedness in effectively handling such situations as an MR, despite their engagement in other educational activities provided in the IMT curriculum, such as lecture-style teaching, self-directed learning, and RWCP.

“I think for the acute SIMs, more of them would be useful. I don't think it's enough to just have one day over two years.” [CT1]

Nevertheless, a small number did express an interest in additional SBE sessions that aim to enhance their communication skills. As many of the participants in the FGs earned their medical qualifications abroad and may not be native English speakers, they might find value in further educational initiatives aimed at enhancing their ability to communicate effectively with patients and colleagues. IMY3 trainees believed that some SBE sessions they participated in would have been more beneficial if received before their transition.

“I wish I had IMPRESS in IMT two, not IMT three.” [MR1]

However, they acknowledged the quality of this educational resource diminishes if many sessions are held in the IMY1 year with limited SBE sessions available overall in the training program. At the core of this notion was the belief that, in general, there is a lack of SBE sessions taking place in IMY2, just prior to making the transition to the MR role. A possible explanation for this may be that the current role of SBE in the IMT curriculum does not focus on maximizing the preparation of trainees for transition or does not prioritize transition as its central goal. This is supported by the notion made earlier that participants feel the acute/emergency scenario SBE sessions currently being delivered are not particularly suited to the complex and advanced encounters they believe they need to be prepared for as an MR in RWCP.

In addition, participants believe that for SBE sessions to be effective, they should be conducted not only regularly and consistently throughout the IMT training program, but also with a more even distribution to maximize their impact. This belief may be rooted in the necessity for ongoing practice of skills and scenarios undertaken in SBE. Trainees worry that without regular practice, the benefits of SBE may be lost, making its educational value questionable. This is in line with the view that RWCP does not adequately provide regular opportunities for participants to maintain the knowledge, skills, and behaviors taught in SBE.

Theme Five: Feedback

The topic of feedback was frequently discussed among the participants. They emphasized the need for consistent, high-quality feedback in order for SBE to effectively serve as a valuable teaching tool. It was acknowledged that adequate time must be set aside during these sessions for SBE facilitators to develop the constructive feedback trainees are seeking.

“…without good quality feedback, the SIM, a lot of the learning from the SIM is lost,…” [MR2]

When participants considered previous SBE sessions valuable and beneficial, high-quality feedback was cited as a key contributing factor to their success.

“What made you think it was useful?” [Facilitator]

“…she gave us feedback individually on the procedure that was done….” [CT6]

However, participants often find feedback in SBE sessions inadequate and seek improvement. They feel that the lack of clear, high-quality feedback hampers the ability of supervisors and trainees to identify areas of concern. This is important as these SBE sessions may be able to identify trainees who might be struggling to meet the standards expected of them from their training program. Identifying struggling trainees promptly is crucial to prevent long-term consequences to trainees and ensure quality patient care.

This concern is further exacerbated by the perception that RWCP does not consistently provide opportunities for feedback or reflection on practice. It is acknowledged that, due to the busy schedules of medical professionals, the various demands on their attention and time, and the lack of constant visible supervision from senior colleagues, feedback and reflection - especially with other members of the healthcare team - often suffer.

Several factors have been identified as potential explanations for the dearth of constructive feedback in SBE. One factor is the diverse cultural values among SBE facilitators, which can influence their perspectives. The different backgrounds of SBE facilitators may result in varying opinions on what constitutes valuable feedback. Consequently, while many may believe they are providing beneficial feedback, some may not view this feedback as constructive. In cases where the feedback is given verbally, trainees may feel reluctant at the time to inquire further, seek clarification, or question the feedback being received.

Another factor is the possible apprehension SBE facilitators have over facing complaints, upsetting trainees, or receiving negative SBE course feedback. This fear may stem from their own past experiences or from stories shared by their colleagues. Because of this worry, mentors may decide to offer feedback that is less likely to trigger an adverse reaction from trainees. It is recognized that when feedback emphasizes areas of performance that are unsatisfactory, some people react emotionally, take criticism personally, or become defensive. As a result, instructors may either ignore areas where trainees underperform or minimize their significance.

“Fear of complaint. I've asked somebody directly about this... I asked them to be hard on me when giving me feedback, and then they opened up to me and told me that generally people don't take it well and complain or give negative feedback on the course.” [MR1]

One more possible explanation is the heavy emphasis on positive feedback, which is frequently perceived as easier to provide than constructive feedback. This could be a reason why SBE sessions often lead to an abundance of positive feedback that many participants believe offers little practical value for their development. The difficulty of providing negative feedback in front of others, especially IMT colleagues, may contribute to this trend, as facilitators may be concerned about the potential impact on trainees' self-esteem.

Participants noted the challenging nature of delivering high-quality feedback, highlighting the pivotal role that training can play. Providing constructive feedback effectively entails thoughtful consideration of language, tone, and method of feedback delivery, as well as the need for specificity of feedback to avoid vagueness, all while being able to critically evaluate a trainee's performance. These skills can be developed through the provision of training for SBE facilitators.

“…it's easier to give some generalized, nonspecific, partially positive feedback than to be specific and tell people how they need to improve.” [MR2]

Although concerns were raised about the feedback from SBE facilitators, the simulated patients received a more favorable mention. These participants were seen as valuable in offering feedback on the non-technical aspects of a clinical encounter. This is understandable, given that many of them lack medical training and can therefore provide valuable insights on areas such as doctors' communication style, empathy, use of simple language, and whether patients' concerns were appropriately addressed. Their valuable feedback was hypothesized to have been influenced by past healthcare experiences, motivating them to improve medical education by being clear about strengths, weaknesses, and suggestions for improvement.

Theme Six: Draw on Others' Experiences

A number of participants believe that the utilization of SBE in other industries, medical specialties, and sectors is more effective. They advocate for the adoption of strategies and approaches akin to those used by these other organizations to enhance the delivery of SBE in the IMT curriculum.

“…we should learn from other, other programmes and other vocations who utilise simulation a lot.” [MR2]

As a high-risk industry, known for its challenging and potentially life-threatening situations, the aviation sector was singled out as an example to emulate. Participants are of the opinion that professionals in this field are better equipped for their positions due to the extensive and rigorous undertaking of SBE before participating in any high-risk activities.

“I mean, aviation has minimum hours of SIM before you even try and go anywhere near an actual plane.” [MR1]

The absence of a similar focus on SBE in the IMT curriculum was raised. It is widely believed that many of the tasks and activities carried out by medical personnel are equally high-risk, and the potential for elevated patient mortality and morbidity rates, patient safety incidents, complaints, and negligence can occur when tasks and activities are performed by inadequately trained individuals or ill-prepared and ineffective healthcare teams. It is therefore believed that a similar approach to that taken by the aviation industry is necessary for developing the efficient and effective multidisciplinary healthcare teams needed to deliver high-quality patient care. In medicine, it was recognized other specialties engaged in more SBE.

“…the surgeons, they do a phenomenal amount of simulation in their surgical training.” [MR2]

However, this view was balanced against the knowledge that these specialties, such as obstetrics and surgery, are far more procedure-orientated. SBE is acknowledged as an effective approach for educating novices on the fundamental concepts of practical procedures, hence it is unsurprising that they play a more prominent role in their training curricula. Nonetheless, they noted these specialties valued the role of SBE in producing higher caliber trainees who are more adept at swiftly transitioning into independent medical practice.

Moreover, there is a feeling that the SBE provided in these specialties is designed to more closely resemble the scenarios in RWCP they were expected to encounter. This helps trainees in these specialties better familiarise themselves with the clinical encounters they may experience, allowing them to have some level of prior experience in handling such situations, even if it is in a simulated environment.

Additionally, participants feel that other medical specialties prioritize the thorough training of individuals for the relatively rare yet high-stakes scenarios they may face in RWCP more than the IMT curriculum does, as demonstrated by their more comprehensive utilization of SBE.

Participants believe that other specialties have recognized the importance of regular practice in skills and scenarios to nurture the development of their trainees and ensure that they meet the necessary standards, as well as the significant role of SBE in facilitating such regular practice. Participants believe that the IMT curriculum should better recognize the importance of repeated practice and not solely rely on RWCP to provide it.

There was also a recognition that further enhancing SBE in this curriculum can be accomplished by being more receptive to the wide range of diverse opinions and ideas from the National Health Service (NHS) workforce. This idea lies in the recognition that the NHS is among the most culturally diverse establishments worldwide. Participants feel that the diverse perspectives of the NHS are underutilized, and there is a failure to fully engage individuals from the wider workforce who want to improve medical education and training but lack the appropriate platform to meaningfully contribute.

## Discussion

Implications for practice

This work builds on existing literature to provide a more comprehensive insight into the perceptions of IMT trainees regarding the role of SBE in aiding their transition to the MR role and its current delivery in the IMT curriculum.

Participants have shown a clear inclination to further practice the knowledge, skills, and behaviors acquired during their SBE sessions. This includes a request for not only more frequent SBE sessions but also access to local teaching facilities to utilize SBE equipment and space. These aspirations are grounded in the belief that consistent practice is vital for preserving the teachings received in SBE. Participants acknowledged that RWCP does not consistently offer the necessary means and opportunities for repeated practice. Studies demonstrate that repetition is fundamentally important for reinforcing the skills acquired through SBE, and neglecting this has been proven to result in skill decline following the completion of SBE sessions [16,17].

Findings regarding the critical role of high-quality feedback, the need for facilitators to be sufficiently trained, and the importance of ensuring that learners feel comfortable and supported during the feedback process are consistent with previously published work [16,17]. The perception among participants that this vital component of SBE is not always performed to the expected level raises significant concerns and diminishes one of the key resources available to IMT trainees during the transition process.

Evidence suggests that, given time constraints, relying exclusively on verbal feedback does not enable the feedback provider to adequately reflect on the student's performance [18]. On the other hand, other alternatives, including the provision of high-quality written individual feedback that may require meetings between faculty members to review each student's performance, have been regarded as time-intensive and not consistently viable [19]. This may account for the deficiencies in feedback that participants have noted in previous SBE sessions they have attended.

The barriers highlighted by participants that hinder SBE facilitators from delivering constructive and candid feedback, including the tendency for feedback to be overly generalized and the apprehension of offending colleagues, have been previously documented in the literature [20]. Should resources permit, offering high-quality, written anonymized feedback that has received consensus from multiple facilitators could help mitigate these perceived barriers. In addition to ensuring that all facilitators undergo thorough training to provide effective feedback as specified by participants, SBE sessions may strive to align with one of the established feedback models. While a critical evaluation of these feedback models is beyond the scope of this study, it is relevant to note that many of these models are intended to address barriers to effective feedback. For example, the Pendleton model encourages the facilitator to determine if the learner is prepared and open to receiving feedback [20].

Numerous trainees experience significant anxiety when transitioning to the MR role, as a significant number of CTs report that they have never before handled referrals, provided guidance to other specialties, or supervised colleagues [21,22]. A proposed solution from participants that could address this issue, while also providing a means for regularly assessing their practice and receiving feedback on their performance, is to formally incorporate a structured shadowing period into the IMT curriculum. This establishes the vital safety net for patients that these trainees desire while enabling them to engage in those unfamiliar tasks that they are expected to perform as an MR, which are not typically carried out by CTs. On the other hand, options like shadowing or "step-up" programs have been proposed in the past and have often been regarded as costly, requiring significant rota planning for both trainers and trainees [21].

For comparison, a compulsory shadowing program currently exists for newly qualified doctors entering their first year of the UKFP. Research by Violeta et al. in 2021 evaluated the effect of this shadowing program for new UK medical graduates, which was implemented in 2012, on seven-day in-hospital mortality rates [23]. The findings indicated no significant correlation between the introduction of the program and in-hospital mortality [23]. This potentially reinforces the argument against committing considerable resources to an equivalent initiative in IMT. However, the study's limited power suggested that a minor impact on mortality could not be entirely excluded [23]. Furthermore, the current literature does not adequately address the perceptions of CTs regarding shadowing and its potential influence on the transition to the MR role, nor its potential effect on patient care. This highlights a potential area of focus for future research.

Notwithstanding the identified limitations in current SBE delivery within the IMT curriculum, participants expressed strong conviction regarding the value of SBE as an educational tool. This value is supported by existing literature, as evidenced by a meta-analysis performed by Chernikova et al. in 2020, which recognized SBE as one of the most effective methods for improving the acquisition of complex skills [24]. This assertion is further reinforced by the perspective expressed by participants, who believe that leaders involved in the design and implementation of the IMT curriculum should strive to align SBE delivery more closely with practices observed in other medical specialties such as surgery or industries such as aviation. The increasing emphasis on and utilization of SBE in surgical training and aviation has positively influenced outcomes. It has been observed that surgeons demonstrate comparable performance on simulators to their performance in actual clinical environments, leading to notable improvements in operative performance and a significant decrease in operative times [25,26]. Whereas the aviation sector transitioned from a safety classification of "risky" in the late 1950s to one deemed "safer" within just a few years following the introduction of SBE [27].

Although participants expressed the view that SBE is not being effectively optimized within the IMT curriculum and is more effectively utilized in other contexts, there was a shared understanding that SBE has inherent limitations and cannot be relied upon exclusively to support the transition process. The predominant sentiment was that finding the optimal use of all available teaching resources represents the most promising approach to pursue. The literature has previously highlighted the notion that SBE cannot represent RWCP in its entirety, is costly, and poses challenges to substantially integrate into a curriculum [28]. Additionally, it has been acknowledged that SBE might be perceived as intimidating or overwhelming for learners, a sentiment expressed in the FGs [29]. Apart from the disappointing outcomes associated with shadowing and the difficulties and resources required to provide effective feedback previously noted, it has been observed that RWCP often fails to provide the necessary opportunities for feedback, debriefing, and reflection, all of which are vital components of the learning process [30]. This emphasizes the critical need to enhance and ultimately optimize each educational tool in the IMT curriculum, recognizing their individual strengths and weaknesses more effectively. Participants perceive that current strategies are failing to adequately address the shortcomings associated with these tools.

Strengths

Several steps were taken to enhance the trustworthiness of the research conducted. This study involved prolonged engagement with the gathered data and peer debriefing with fellow medical education research supervisors to ensure an external review of the research process [13,14]. Additionally, cross-referencing data from various time periods and groups of people by undertaking multiple FGs, and involving other researchers in data analysis to achieve investigator triangulation, were undertaken [13,14]. The purpose of these steps was to enhance the reliability and objectivity of the findings by reducing the researchers' preconceived notions and biases related to the topic of study. This is of particular importance given that the researcher who conducted the FGs and TA of the data collected is a doctor who completed the IMT program and transitioned to the role of MR through this program.

The study employed both convenience and criterion sampling methods. While this approach may not have ensured representativeness, it aligns with the principles of phenomenological research, which prioritize conceptual requirements over representativeness in sample selection [15]. This sampling approach attempted to select participants with the relevant individual experiences and range of characteristics in sufficient numbers to gather the information needed for a thorough understanding of the phenomenon being studied [15].

Limitations

Owing to practical difficulties, the decision to focus recruitment of IMT trainees on a single region, rather than engaging in a national recruitment effort, represents a limitation, as it resulted in the omission of valuable perspectives and insights from other NHS deaneries.

Another notable limitation is the inadequate number of participants who took part in the FGs. Several factors may have contributed to this unfortunate outcome. Regrettably, the FG sessions occurred during a period when many IMT trainees were engaged in PHST applications, which likely diverted their attention toward preparing for interviews. Numerous IMT trainees may have chosen not to participate owing to various obligations, such as preparing for postgraduate examinations, developing ePortfolios for end-of-year appraisals, or becoming occupied with their daily work responsibilities, despite their initial interest in attending. It is also important to highlight the junior doctor industrial strike action that took place, which may have influenced engagement levels. IMT trainees may have utilized this time to catch up on training requirements delayed by the strikes.

## Conclusions

This study reinforces the value of SBE and its essential contribution to contemporary medical education, its role in the transition process, and its perceived success in other medical specialties and industries. It identifies some fundamental principles that IMT trainees believe are necessary to optimize its educational potential, thereby allowing the IMT curriculum to better meet one of its primary objectives: to adequately support the transition to the MR role, which IMT trainees believe is not being effectively realized. IMT trainees recognize the limitations of SBE. As a result, they have called for more concerted efforts to enhance the utilization of all educational tools present in the IMT curriculum, which include feedback, RWCP, shadowing, and SBE. This stems from the conviction that no single educational tool can be regarded as a comprehensive solution to transition.

It was evident that IMT trainees sought greater opportunities for continuous application of the knowledge, skills, and behaviors acquired in SBE. This need was communicated in several ways, including requests for additional SBE sessions, more effective scheduling of these sessions, and improved access to resources to facilitate independent practice. These findings indicate that stakeholders involved in the design and implementation of the IMT curriculum should seek to further explore the effectiveness of the current IMT program in preparing trainees to transition, as well as evaluate the quality of the support structures currently established to facilitate this difficult and challenging phase. Further research is needed into the effectiveness of a formalized shadowing program for CTs, the nature, perspectives, and challenges of transitioning from CT to MR as well as the views and insights of IMT trainees from all deaneries and regions throughout the UK.

## Figures and Tables

**Figure 2:**
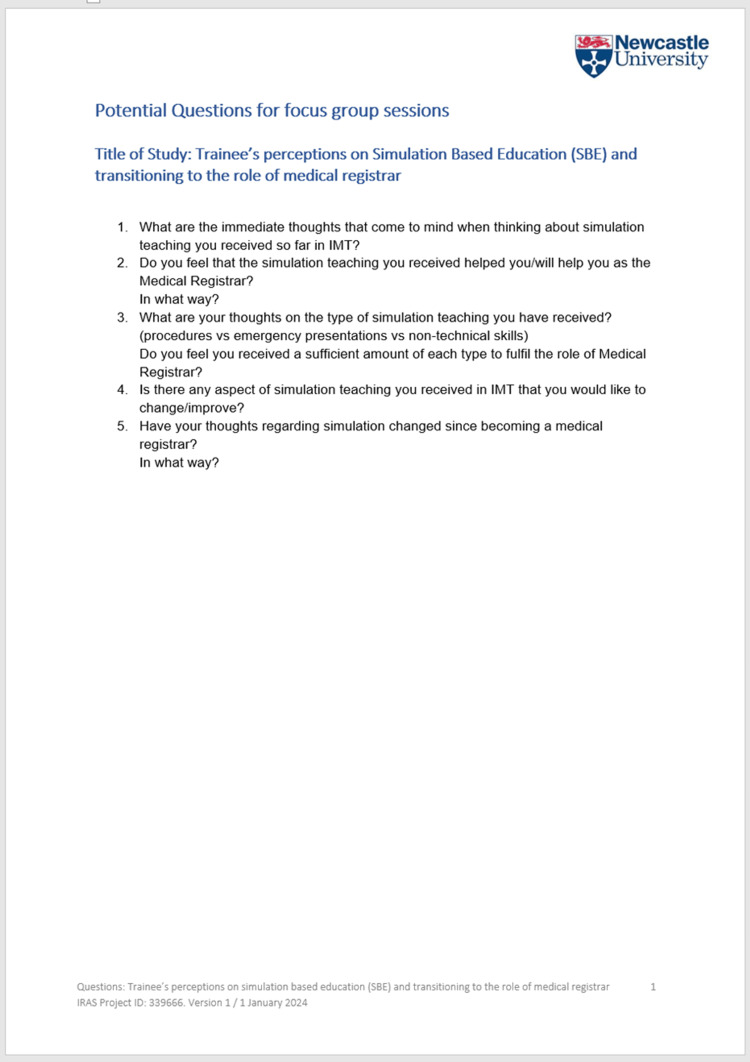
Set of facilitator questions.

**Figure 3:**
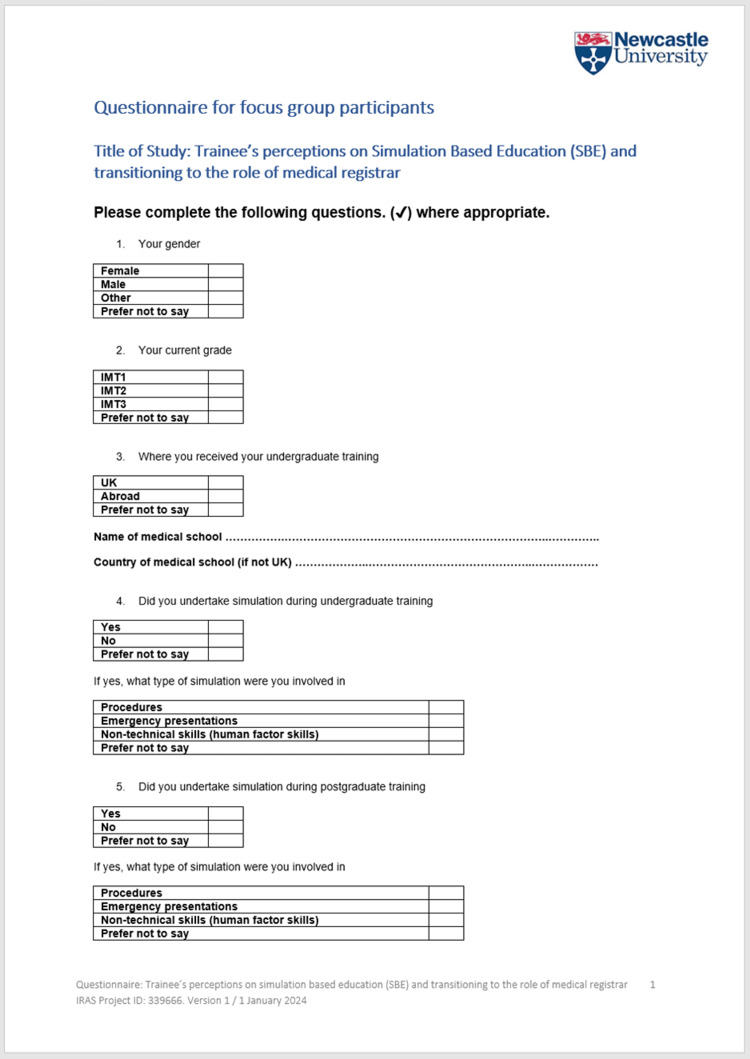
Demographic data and participants' previous experience questionnaire.

## Appendices

Appendix A

Appendix B 
